# Suicidal ideation and its correlates among high school students in Iran: a cross-sectional study

**DOI:** 10.1186/s12888-017-1298-y

**Published:** 2017-04-20

**Authors:** Reza Ziaei, Eija Viitasara, Joaquim Soares, Homayoun Sadeghi-Bazarghani, Saeed Dastgiri, Ali Hossein Zeinalzadeh, Farhad Bahadori, Reza Mohammadi

**Affiliations:** 1 Department of Health Sciences, Unit for Public Health Science, Mid Sweden University, Sundsvall, Sweden; 2 Road Traffic Injury Research Center, Department of Statistics & Epidemiology, Tabriz University of Medical Sciences, Tabriz, Iran; 3 Department Of Community Medicine, Tabriz University of Medical Sciences, Tabriz, Iran; 4 Department of Health Education and Promotion, Faculty of Medical Sciences, Tarbiat Modares University, Tehran, Iran; 5 Department of Neurobiology, Care Sciences and Society, Unit for Family Medicine, Karolinska Institute, Stockholm, Sweden

**Keywords:** Suicidal ideation, Suicide, Adolescents, High school students, Global School-Based Student Health Survey, Iran

## Abstract

**Background:**

Globally, the second leading cause of death among adolescents is suicide and in middle-income countries adolescents’ suicidal ideation is a neglected public health area. The present study was conducted to determine the prevalence and correlates of suicidal ideation among 15–17-year-old high school students in Iran.

**Methods:**

Self-administered, Global School-based Student Health Survey (GSHS) questionnaires were distributed to a representative sample (*N* =1517) of high-school students aged 15–17 in the city of Tabriz. Multivariate logistic regression was used to assess the association between relevant independent variables (e.g. gender) and the dependent outcome variable (suicidal ideation in the past 12 months).

**Results:**

Overall, 62 (4.1%, 95% CI= 3.1, 5.2) of 1,517 students had thoughts of suicide. Three hundred and thirteen (20.6%, 95% CI= 18.6, 22.7) students reported being bullied in the previous 30 days. In addition, 134 (8.8%, 95% CI= 7.5, 10.3) students reported having been sexually abused. Being worried that they could not eat or did not feel hungry (Adjusted Odds Ratio (AOR) = 4.15; 95% Cl [1.71, 10.07]; current cigarette smoking (AOR = 3.00; 95% CI [1.69, 5.30]; thinking about using alcohol or other drugs (AOR = 4.28; 95% CI [2.41, 7.59]; and being sexually abused (AOR = 2.63; 95% CI [1.32, 5.24]) were all factors positively associated with suicidal ideation.

**Conclusion:**

The prevalence of suicidal ideation was lower in our school students than in earlier studies. Interventions that address the issue of current cigarette smoking, worries, thinking about using alcohol or other drugs and sexual abuse should be given more priority by the public health authorities.

## Background

Globally, the second leading cause of death among young people aged 15-29 years is suicide. In 2012, suicide was the cause of 804,000 deaths worldwide, and since it bears a stigma in most countries, it is very likely that cases are under-reported [1].

Suicidal behavior has different stages, such as suicidal ideation, suicide planning, and attempting suicide [2]. Wishes, ideas, and the tendency towards committing suicide are defined as suicidal ideation [3]. In adolescents, suicidal ideation has been reported as an important risk factor for suicide [4]. It is also associated with a subsequent risk of attempting suicide [5]. In the general population, a suicide attempt is the most important risk factor for suicide, and the risk of suicide increases with the number of attempts [6]. According to a systematic review, around one third of adolescents aged 12-20 years have reported suicidal ideation [7].

Adolescence is a transitional stage from childhood to adulthood and during this period, adolescents experience many changes. These include physical growth, new social relations, and also emotions that can put a great deal of pressure on them. Without social and parental support, this pressure can put adolescents at risk of suicide [8, 9].

Many factors can lead an adolescent to suicidal behavior, and these can be divided into two categories: psychological problems (loneliness, worry, hopelessness) [10–12]; and social-environmental factors such as low, or lack of, parental or peer support**,** harmful alcohol and drug use, smoking [11, 13–15], and being bullied or sexually abused [11, 16, 17]. As mentioned, psychological problems and social-environmental factors related to an individual’s life history are the main risk factors for suicide [8, 18]. In contrast, parental support, supervision, understanding adolescents’ problems and worry, and peer support at schools have all been recognized as protective factors against suicidal ideation [19].

**Fig. 1:**
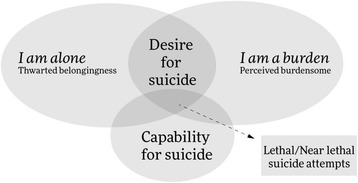
Interpersonal Theory of Suicide

From a theoretical aspect, suicidal behavior can be explained by different theories (e.g., biological), and each theory can clarify one or more aspects of suicidal behavior. A theory that seems appropriate in explaining suicidal behavior and risk factors is the interpersonal theory of suicide. According to this theory people commit suicide because they want to and are able to (see Fig. 1)**.** It also proposes three important components that lead a person to suicidal behavior: thwarted belongingness and perceived burdensomeness, both of which are related to suicidal desire and, thirdly, suicide-related capability. The simultaneous presence of the first two components leads the person to want to commit suicide, whilst the third component empowers the person to commit suicide without fear of death. People must acquire the capability to commit suicide. The more often a person attempts to commit suicide, the more its taboo and the fear and pain associated with self-harm diminish and consequently the easier it is to commit suicide. In this theory, some risk factors for lethal suicide are empirically described, such as mental disorders, previous suicide attempts, social isolation, family conflict, childhood abuse, hopelessness, etc. [20–22]. Feeling lonely (thwarted belongingness), and a sense that one is a burden on others (feeling perceived burdensomeness) are sufficient causes of passive suicidal ideation and can lead an adolescent to think about suicide**.** As mentioned, feeling lonely is a psychological problem and a risk factor for suicidal behavior. According to the literature, family and peer support can protect adolescents from feelings of isolation and increase their sense of belongingness to family and society, which in turn can act as a protective factor against suicidal ideation [19].

According to the latest Suicide Report of the World Health Organization (WHO), the suicide rate (per 100 000) in 2012 among 15-29-year-olds in Iran for both sexes was 7.8. Broken down by sex, it was 10.00 for males and 5.5 for females [1]. The rate of suicide in the general population varies from province to province. According to the Iranian Forensic Medicine Bureau, the national rate of suicide for males was 5.7 and 3.1 for females per 100,000 but in the western provinces of Iran, the rate fluctuates between 40 and 66 cases per 100,000 [23].

Because of the sensitivity of the topic and the special rules governing the data collection from adolescents in Iran, there are very limited studies on the prevalence and correlates of suicidal ideation among high-school students. There are just a few studies which have been done on community samples of adults [23, 24]. Moreover, no comprehensive standardized questionnaire has been available to survey students’ feeling of loneliness, loss of sleep and appetite due to worry, suicide ideation and attachment to peers. Also, there has been no reliable and valid questionnaire in Persian for gathering data on suicidal ideation among adolescents. The authors designed the present study to bridge this data and instrument gap and used the reliable Persian version of the Global School-Based Students Health Survey (GSHS), a comprehensive questionnaire validated by the authors [25] in a pilot study before the main survey. This was done to determine the prevalence and correlates of suicide ideation and to help design interventions to promote mental health among high school students in Tabriz.

The aim of the present study was to examine whether suicidal ideation among students is positively associated with smoking, bullying, sexual abuse, thinking about alcohol consumption, loneliness and being worried, and negatively associated with parental understanding/support.

## Methods

### Study site and sampling

Iran is a middle-income country in the Eastern Mediterranean Region (EMRO) with a population of approximately 80 million. Tabriz city, where the present cross-sectional study was conducted, is located in northwest Iran and has around 1,700,000 inhabitants. At the high-school stage (grades 9 to 11^th^), there were 62,714 school students, (29,935 girls and 32,779 boys) during the 2013-14 academic years. Two-stage cluster sampling was used to select representative high-schools and classes. At the first stage, high-schools were selected with a probability proportional to the enrollment size and at the second stage classes were randomly selected. All students in the selected classes were then eligible to participate in the study. Overall, thirty high-schools, including sixteen girls’ and fourteen boys’ high-schools, and ninety classes (grades 9 to 11^th^) [26] were chosen to participate in the study. The full details of the methodology have been reported earlier [27].

### Participants

The study participants included 727 (47.9 %) males and 790 (52.1 %) females. Students’ ages ranged from 15 to 17 with a mean of 16.1 ± 0.76. Four hundred and thirty-five (28.7 %) students were from grade 9, 539 (35.5 %) from grade 10, and 543 (35.8 %) from grade 11. Up until the high-school diploma, the Iranian education system comprises three stages: stage one or elementary school, which includes 5 grades ^(1-5th)^; stage two or guidance school, which includes 3 grades ^(6-8th)^; and stage three or high school, which includes 3 grades ^(9-11th)^. For the present study, stage-three students were chosen as participants. The pre-university stage includes just 1 grade ^(12th)^and is applicable for students that want to enter university, otherwise at the end of the high-school stage, the high-school diploma is awarded to the students [26].

### Data collection and tools

An anonymous, self-administered, validated and reliable Persian Version of the GSHS Questionnaire for Mental Health was used for data collection [25, 28]. For the reliability and validity of the Persian version GSHS questionnaires, the original English GSHS Core and Core- Expanded versions were translated into Persian and translated back into English. The English versions were then compared and, in order to ensure consistency between the two versions, the Persian version was then edited. For internal consistency (Cronbach's α), test-retest reliability and Spearman’s correlation between total test and re-test scales score were used. The Content Validity Index (CVI) was used to address validity. The questionnaires were then pilot-tested and revised for clarity, the full details of which have been reported earlier [25]. The reliable and validated Persian version module comprised 12 questions, two of which were related to suicide attempts and plans and were deleted by the authorities because of the sensitivity of the questions. This left only one question relating to considering suicide (suicidal ideation) in the questionnaires. As a result, the questionnaires were supplemented with other questions about risk factors for suicide ideation which were found in our literature review. The final mental health module of the main survey includes 14 questions in two sections: the first section has nine questions regarding students’ mental health status and the second section has five questions about knowledge, attitudes, skills, and sources of information (see Table 1). According to the WHO’s GSHS item rationales, the mental health questionnaire measures feelings of loneliness, loss of sleep due to worry, sadness and hopelessness, suicide ideation and attempts, and attachment to peers [29].

**Table 1:** Characteristics of the students (*N*= 1517) in the GSHS Mental Health Module, 2012-13

Item	Males (no/%)	Females (no/%)	Total (no/%)
During the past 12 months, have you felt lonely?	(589/81.0)	(699/88.5)	(1288/84.9)
During the past 12 months, have you been so worried about something that you could not sleep at night?	(534/73.5)	(600/75.9)	(1134/74.8)
During the past 12 months, have you ever seriously considered attempting suicide?	(27/3.7)	(35/4.6)	(62/4.1)
During the past 12 months, have you been so worried about something that you wanted to use alcohol or other drugs to feel better?	(114/15.7)	(86/10.9)	(200/13.2)
During the past 12 months, have you been so worried about something that you could not eat or did not feel hungry?	(432/59.4)	(462/58.4)	(894/58.9)
During the past 12 months, have you had a hard time staying focused on your homework or other things you had to do?	(534/73.5)	(571/72.3)	(1105/72.8)
Have you been bullied during the past 30 days?	(193/26.5)	(120/15.2)	(313/20.6)
Have you ever been sexually abused?	(58/8.0)	(76/9.6)	(134/8.8)
During the past 30 days, did your parents or guardians understand you?	(552/75.9)	(576/72.9)	(1128/74.4)
During this school year, were you taught in any of your classes how to manage anger?	(112/15.4)	(94/11.9)	(206/13.6)
During this school year, were you taught in any of your classes about the signs of depression and suicidal behavior?	(53/7.3)	(93/11.8)	(146/9.6)
During this school year, were you taught in any of your classes about what to do if a friend is thinking about suicide?	(52/7.2)	(87/11.0)	(139/9.2)
During this school year, were you taught in any of your classes how to handle stress in healthy ways?	(93/12.8)	(159/20.1)	(252/16.6)
Have you smoked cigarettes during the past 30 days?	(146/20.1)	(83/10.5)	(229/15.1)

Data collection for the present study took place between December 2013 and February 2014. Self-administered questionnaires were distributed to students in selected schools and classes during an ordinary school day by researchers with the help of Province Health Center staff who, one week prior to administration date, had attended a workshop on GSHS methodology. GSHS is a self-administered, school-based questionnaire for monitoring students’ health status. It has been designed by the WHO in collaboration with UNICEF, UNESCO and with the technical support of the United States Center for Disease Control and Prevention (CDC) as a global, youth health monitoring surveillance system. The Province Health Center is the responsible authority for the health of school students and mediates between Tabriz Medical University and the Ministry of Education, which is responsible for schools. Answering time for questions was around 20 min.

### Data processing and analysis

#### Measure

Suicidal ideation was our dependent outcome variable and was addressed by the question: “During the past 12 months, have you ever seriously considered attempting suicide?”

Feeling lonely was addressed by the question: “During the past 12 months, have you felt lonely?” Smoking cigarettes was addressed by the question: “Have you smoked cigarettes during the past 30 days?” The wish to use alcohol and other drugs was addressed by the question: “During the past 12 months, have you been so worried about something that you wanted to use alcohol or other drugs to feel better?” Being bullied was addressed by the question: “Have you been bullied during the past 30 days?” Sexual abuse was defined as “Having sexual intercourse against one’s will.” and was addressed by the question: “Have you ever been sexually abused?” The presence of understanding parents was addressed by the question: “During the past 30 days, do you feel that your parents or guardians have understood you?” The answers were dichotomized in a Yes/No format.

#### Statistical analysis

The schools’ response rate was 100% and the students’ response rate 99.08 %. During data collection, 1,531 questionnaires were distributed to students but only 1,517 questionnaires were returned to staff. SPSS version 23 was used for data entry and analysis, with the STATA 13 statistical software package being used to derive the beta-weights. Descriptive statistics were used to analyze categorical and continuous scales, the Chi square, and Fisher exact tests to assess associations among categorical variables. A multivariate logistic regression was used to assess the association between relevant independent variables (e.g. cigarette smoking) and the dependent outcome variable (suicidal ideation). Reporting of adjusted odds ratios (AORs) and unadjusted odds ratios (UAORs) was done after controlling for factors identified as significant in the bivariate analysis. AORs and UAORs with 95% confidence intervals (CI) were reported. Through the pre-modeling bivariate analysis, associations with *p*-value < 0.1 were considered for further analysis as an independent variable in multivariate analysis. A *p*-value of less than 0.05 was considered statistically significant for other statistical tests.

## Results

### Prevalence of suicidal ideation and other factors

As shown in Table 1, 62 (4.1%, 95% CI= 3.1, 5.2) students mentioned that they had seriously considered attempting suicide in the past 12 months. Additionally, 1,288 (84.9, 95% CI= 83.0, 86.6) students stated that they had felt lonely and 200 (13.2, 95% CI= 11.5, 14.9) reported that they had been worried and had wanted to use alcohol or other drugs. Bullying was relatively common, and 313 (20.6%, 95% CI= 18.6, 22.7) students complained of being bullied during the past 30 days. In addition, 134 (8.8%, 95% CI= 7.5, 10.3) students reported having been sexually abused. Another 389 students (25.6%, 95% CI= 23.6, 27.9) stated that their parents did not understand them (their problems). Finally, 229 (15.1%, 95% CI= 13.3, 16.9) students reported that they had smoked cigarettes during the past 30 days (current smoker).

### Factors associated with suicidal ideation

As shown in Table 2, in the UAORs, suicidal ideation was positively associated with being worried (UAOR = 6.94; 95% Cl [2.97, 16.23], struggling to stay focused on homework (UAOR = 3.07; 95% Cl [1.38, 6.80], current cigarette smoking (UAOR = 4.81; 95% CI [2.85, 8.13], thoughts about using alcohol or other drugs (UAOR = 7.69; 95% CI [4.55, 12.98], being bullied (UAOR = 2.54; 95% CI [1.50, 4.31], and being sexually abused (UAOR = 3.28; 95% CI [1.75, 6.13]). Suicidal ideation was negatively associated with having understanding parents (UAOR = 0.49; 95% CI [0.29, 0.83]).

**Table 2:** Correlates with suicidal ideation among high-school students in Iran

Unadjusted odds ratios with 95% CI			Adjusted odds ratios with 95% CI			
Bivariate analysis			Multivariate analysis			
Independent variables	UAOR	CI 95%	AOR	CI 95%	b	bStdX
Age^c^	1.23	0.73 – 1.43				
Gender^a^						
* Female*	1.21	0.72 – 2.02				
* Male* ^*g*^	1.00					
Grade^a^						
* Second* ^*e*^	0.70	0.36 – 1.37				
* Third* ^*f*^	1.09	0.59 – 2.00				
* First* ^*g*^	1.00					
Feeling lonely	2.11	0.83 – 5.32				
Being worried, that you could not eat or did not feel hungry	6.94	2.97 – 16.23****	4.15	1.71-10.07**	1.42	0.70
Having a hard time to stay focused on homework	3.07	1.38 – 6.80**	1.44	0.62-3.37	0.36	0.16
Smoking (current)	4.81	2.85 – 8.13 ****	3.00	1.69 – 5.30****	1.09	0.39
Ideation to use alcohol or other drugs	7.69	4.55 – 12.98****	4.28	2.41 – 7.59****	1.45	0.49
Being bullied	2.54	1.50 – 4.31 ***	1.43	0.81 – 2.53	0.36	0.14
Sexually abused	3.28	1.75 – 6.13****	2.63	1.32 – 5.24***	0.96	0.27
Understanding parents	0.49	0.29 – 0.83**	0.66	0.37 – 1.17	- 0.40	- 0.17

*Cl* Confidence interval, *OR* odds ratio, *bStdX* standardized beta coefficients

*p<0.05; **p<0.01; ***p<0.001; ****p<0.0001, R^2^ = 0.000

^a^categorical variables, ^b^b coefficients; ^c^Continuous variable; ^d^First level of high school; ^e^second level of high school; ^f^Third and final level of high school; ^g^Comparison group

However, in the AOR, only being worried (AOR = 4.15; 95% Cl [1.71, 10.07], current cigarette smoking (AOR = 3.00; 95% CI [1.69, 5.30], thoughts about using alcohol or other drugs (AOR = 4.28; 95% CI [2.41, 7.59] and being sexually abused (AOR = 2.63; 95% CI [1.32, 5.24]) remained positively associated with suicidal ideation.

## Discussion

### Prevalence of suicidal ideation

The results of the present study show that 4.1% of the students had seriously considered attempting suicide during the past 12 months. We could not find any study conducted among high-schools in Iran to make a comparison. However, in some regions of Iran (e.g. the Western provinces), the prevalence of suicide ideation and suicide attempts appears to be high [23]. Additionally, according to a study conducted in Karaj city (Iran), the prevalence of suicide ideation, suicidal plans and suicide attempts among the general population was 12.7, 6.2 and 3.3%, respectively [24].

Some comparisons can be made between our study and those from other countries using data derived from GSHS surveys. For instance, among Brazilian adolescents aged 13-18 years, the prevalence of suicidal ideation was 14% [30], in Zambia it was 32.2 % in school adolescents [14] and in Guyana it was 18.4 % [31]. Furthermore, in Tanzania the rate of suicidal ideation among adolescents was 11.2 % [12] and in school-going adolescents in Thailand 8.8 % [32]. In GSHS surveys among EMRO countries, the prevalence of suicidal ideation varies from 13 % - 17 % [11].

Compared with other countries, therefore, the rate of suicidal ideation in the city of Tabriz is lower, and this low rate may be explained by the provincial culture encouraging strong family bonds which may provide a protective effect [11, 14, 33] among adolescents. The family may also be able to provide support and help to its members during hard times [34]. Religious thinking, which plays an important part in the life of people in Islamic countries, may have a strong role. In Islam, self-killing is denounced and considered Haram (forbidden) and against God [35]. On the other hand, the sensitivity of the topic, it being taboo in society, especially in Islamic culture, and the lack of a reliable method for detecting under-reporting all mean that our figures may be under-estimated.

### Related factors

The students who reported a loss of appetite due to worry had approximately four times higher odds of suicidal ideation than students who were not worried. Comparable results were found in other GSHS reports conducted in Zambia [14] and Venezuela [36]. Worry may be caused by different factors (e.g. socio-economic, educational, poor health) [37]. These should be scrutinized to avoid future problems which may lead to suicidal ideation, suicide attempts and mental health problems among adolescents. For instance, an Iranian study which assessed the number of suicide attempts resulting in death among the general population suggested that a high unemployment rate among young people can cause bewilderment and disappointment about future life and hence be the main risk factor behind suicidal ideation [23].

Our results indicate that current smoking, and thoughts about using alcohol or other drugs to feel better are associated with higher odds of suicidal ideation. Several studies suggest that alcohol and substance abuse are positively associated with suicidal ideation [13, 38]. To the best of our knowledge, there is not enough formal and published information about alcohol consumption among adolescents in Iran due to legal restrictions. Furthermore, a recent study [39] showed a positive association between suicidal ideation and non-medical use of prescription drugs (NMUPD). Another study among school students in the Republic of Benin has reported that alcohol misuse and illicit drug use are associated with suicidal ideation [33]. In a study conducted among 75,643 students from middle and high-school stages in South Korea concerning the association between suicidal ideation and smoking, statistically significant associations were found between smoking and suicidal behavior (ideation, plan, attempts), particularly among those who smoked a higher number of cigarettes per day [40]. Persistent sexual abuse was positively associated with suicidal ideation. Indeed, sexually abused students had 2.63 times higher odds of suicidal ideation. Our findings are in line with studies showing a positive relationship between sexual abuse and suicidal behavior and ideation [41–43]. Furthermore, being sexually abused and negative mental health outcomes, such as depression, smoking and alcohol use, put adolescents at a greater risk of suicidal behavior [44, 45]. The high occurrence of sexual abuse among our students (8.8%) raises many questions; however, it is more difficult to conjecture how the students were abused. This issue needs further research which is outside the scope of the present study and data.

### Multi-victimization

Multivariate analysis showed that the association between bullying and suicide ideation cannot be considered as an independent association, so adding an interaction without a main effect was not considered. The association found through bivariate analysis seems to be a confounded association due to the coincidence of bullying with sexual abuse. Bullying was therefore not considered a risk factor for suicide ideation in this study.

### Study limitations

The present study has some limitations. First, because of the sensitivity of the topic, the authors were not allowed to assess plans to attempt suicide and suicide attempts. Second, it is a cross-sectional study and does not allow causality to be established for any of the associated factors in the study. Third, the students were from one urban center in Iran and they may not be representative of those from other urban centers, non-urban areas and adolescents not attending school. Fourth, despite the anonymous nature of the study, because of the social and cultural taboo about suicidal ideation, participants may have intentionally or unintentionally misreported and/- or underreported when answering the questions.

## Conclusion

This study showed the prevalence and related correlates of suicidal ideation among high-school adolescents in the city of Tabriz. The prevalence of suicidal ideation was lower in our studied school students than in non–Iranian studies. Identified risk factors should be taken into consideration by public health authorities in the development and implementation of interventions aiming at educating students in how to cope with stressful situations to avoid risky behaviors and offer protection against suicide ideation and behavior.

## Abbreviations

AORs: Adjusted odds ratios
CDC: Center for Disease Control and Prevention
CI: Confidence intervals
EMRO: Eastern Mediterranean Region
GSHS: Global School-Based Students Health Survey
NMUPD: Non-medical use of prescription drugs.
UAORs: Unadjusted odds ratios
WHO: World Health Organization
